# Understanding and caring for a Schiotz tonometer

**Published:** 2014

**Authors:** Ismael Cordero

**Affiliations:** Clinical Engineer, Philadelphia, USA. ismaelcordero@me.com

**Figure:**
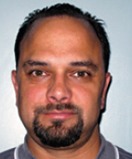
Ismael Cordero

A Schiotz tonometer is an instrument for measuring the intraocular pressure (IOP).

Although the Schiotz tonometer does not make as precise measurements as other types of to no meters, it is inexpensive, simple to use, durable, requires little maintenance, does not have electronics, does not require batteries, and can be stored for years between uses. These qualities make it well suited for screening and remote or mobile clinics.

The Schiotz tonometer consists of a hollow barrel with a concave footplate and a holder (Figure 1). A free-floating, rod-like plunger with a 5.5 gram weight attached fits inside the barrel. When held vertically on top of the eye, the plunger will move downwards by gravity and indent the cornea. This very small up-and-down movement is magnified by a lever arm to move a needle that gives a reading on a horizontal scale numbered arbitrarily 0–20. A firmer eye, due to higher IOP, will result in a lower indentation and a lower reading on the scale.

Since the Schiotz tonometer does not measure pressure directly, a conversion table, supplied with the instrument, is used to translate scale readings into estimates of IOP in mmHg. To account for the range of pressure, other weights (typically 7.5 g and 10 g) are supplied that can be added to the plunger.

**Table 1.** Schiotz scale conversion table

Scale reading	Ocular pressure, mmHg
	5.5 g weight	7.5 g weight	10.0 g weight
**3.0**	24.4	35.8	50.6
**4.0**	20.6	30.4	43.4
**5.0**	17.3	25.8	37.2
**6.0**	14.6	21.9	31.8
**7.0**	12.2	18.5	27.2
**8.0**	10.2	15.6	23.1
**9.0**	8.5	13.1	19.6
**10.0**	7.1	10.9	16.5

**Figure 1:**
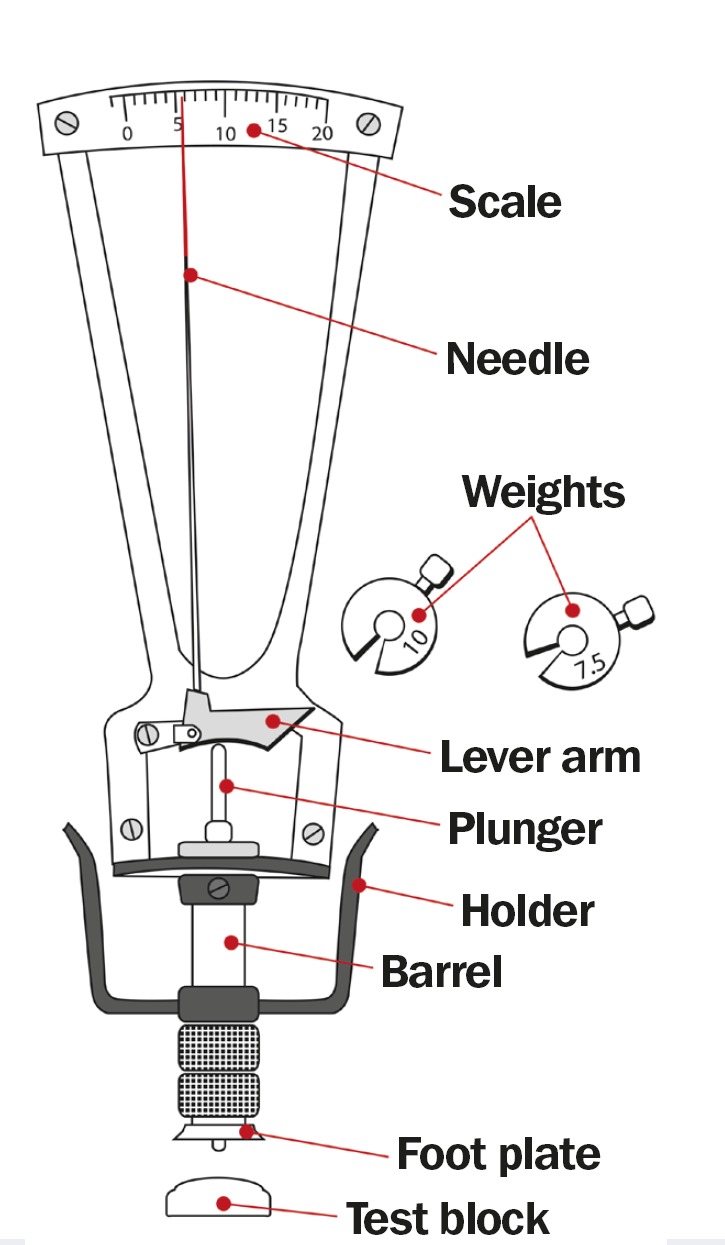


## Calibration check procedure

A calibration check should be done at the start of every day. Place the footplate of the instrument on the rounded test block (the dummy cornea) provided with the tonometer's storage case. With the footplate resting on the test block, a correctly calibrated instrument will have a scale reading of zero.

If not, you can calibrate it to zero.

- If the needle is to the **left** of zero, rotate the footplate in a **clockwise** direction and check again.
- If the needle is to the **right** of the zero position, rotate the footplate in an **anti-clockwise** direction.

## Cleaning

Cleaning of the barrel and the plunger should be done once a day to prevent the plunger from sticking to the barrel.

1. Remove the plunger and use gauze with alcohol to clean the plunger and tip.
2. Clean the inside of the barrel with an alcohol-soaked cotton swab and then with a dry cotton swab.
3. Clean the footplate with gauze and alcohol.
4. Allow it to dry and then place, disassembled, in its case.

In between patients, the Schiotz tonometer should be disinfected by soaking it in sodium hypochlorite.

